# HIV associated immune aging among people living with HIV: implications for long-term antiretroviral therapy and potential HIV cure interventions in sub-Saharan Africa

**DOI:** 10.3389/fimmu.2026.1918867

**Published:** 2026-09-03

**Authors:** Praiscillia Kia, Edward Kankaka, Patricia Kankundiye, Marcel Tongo, Rose Nabatanzi, Tokameh Mahmoudi, Damalie Nakanjako

**Affiliations:** 1 Department of Immunology and Molecular Biology, School of Biomedical Sciences, Makerere University College of Health Sciences, Kampala, Uganda; 2 Infectious Diseases Institute, Makerere University College of Health Sciences, Kampala, Uganda; 3 Africa-Europe Cluster of Excellence in Translational Science in Infection, Immunity and Inflammation (CoRE Triii), Makerere University College of Health Sciences, Kampala, Uganda; 4 Rakai Health Sciences Project, Kalisizo, Uganda; 5 The Centre for Research on Emerging and Re-emerging Diseases (CREMER), Yaoundé, Cameroon; 6 Higher Institute for Scientific and Medical Research (ISM), Yaoundé, Cameroon; 7 HIV Pathogenesis Programme, The Doris Duke Medical Research Institute, University of Kwazulu Natal, Durban, South Africa; 8 Department of Pathology, Erasmus Medical Centre, Rotterdam, Netherlands; 9 Department of Urology, Erasmus Medical Centre, Rotterdam, Netherlands; 10 Department of Medicine, School of Medicine, Makerere University College of Health Sciences, Kampala, Uganda

**Keywords:** antiretroviral therapy (ART), chronic HIV infection, comorbidities, HIV cure, human immunodeficiency virus (HIV) reservoir, immune aging, immune senescence, sub-Saharan Africa

## Abstract

**Background:**

Clinical and biological consequences of accelerated immune aging are substantial, with growing evidence of increased risk of non-communicable diseases (NCD) among people living with HIV (PLHIV). However, there is limited understanding of reliable biomarkers of immune aging, singly or in combination, that can be used for monitoring HIV treatment in large longitudinal cohorts in sub-Saharan Africa (SSA). This review describes candidate biomarkers of immune aging during HIV treatment and their potential clinical application in monitoring immune aging, immune function recovery and risk of NCD complications among adults aging with HIV and ART.

**Methods:**

On April 1, 2026, we searched PubMed using a broad Boolean strategy that combined MeSH terms and title/abstract keywords for HIV-related concepts with MeSH terms and title/abstract keywords for immunosenescence-related concepts. The HIV block included terms such as HIV, HIV infections, HIV seropositivity, HIV-1, HIV-2, AIDS, and descriptors for people living with HIV, while the immunosenescence block included immunosenescence, T cell senescence, cellular senescence, immune aging/ageing, inflammaging, age-associated immune changes, and accelerated or premature immune aging.

**Results:**

Key findings reveal that CD4/CD8 ratio inversion, T-cell senescence markers including CD28 and CD57^+^ expression on CD4/CD8 T-cells and Natural Killers cells, PD-1 expression, as well as selected inflammatory cytokine panels and proteomic signatures of immune aging are promising biomarkers that could contribute to monitoring immune aging during long-term ART. In addition, there is evidence to suggest association of immune aging with HIV reservoir characteristics during ART. However, validation of immune aging markers remains incomplete in SSA populations with chronic HIV infections, host genetic diversity, HIV viral non-B sub-type diversity, variable ART access and multiple endemic co-infections.

**Conclusion:**

This systematic review underscores the need for evidence to develop HIV-associated immune aging biomarker panels during long-term HIV treatment in SSA; to guide the development of predictive, diagnostic and/or monitoring biomarker panels for HIV-associated immune aging and its complications among PLHIV in SSA. We recommend well-characterized human studies to inform the adoption of context-specific HIV cure innovations in consideration of the heterogeneous host immune aging phenotypes, HIV-1 viral sub-types and endemic co-infections in SSA.

## Introduction

Despite long-term antiretroviral therapy (ART), people living with HIV (PLHIV) continue to exhibit persistent immune dysfunction characterized by chronic immune activation, systemic inflammation, and features of accelerated immune aging (1–3). The features of HIV-associated immune aging include expansion of senescent and exhausted T-cell populations, altered CD4/CD8 ratios, impaired immune regeneration, and molecular aging signatures such as telomere shortening and DNA damage (4–6). While these features occur as a natural consequence of chronological aging in a healthy population (7), they appear earlier and more intense in PLHIV hence the term “accelerated immune aging”. The biological and clinical consequences of accelerated immune aging are substantial, with evidence of increased risk of non-communicable diseases (NCD) including cardiovascular, metabolic, neurocognitive, and malignant conditions (8–10). Notably, accelerated immune aging occurs in PLHIV irrespective of virologic suppression, indicating incomplete immune recovery during long-term HIV treatment with protracted inflammation and immune exhaustion that drives immune aging and persistent immune dysfunction. Emerging literature suggests that persistent and transcriptionally active HIV proviruses in HIV-infected cells may be sufficient to drive this residual dysfunction, and are associated with telomere attrition and age-related expansion of infected cell populations, suggesting a bidirectional relationship between immune aging and viral persistence (11–14). Moreover, there is limited understanding of reliable biomarkers of immune aging, singly or in combination, that can be used during routine treatment monitoring in large longitudinal cohorts in sub-Saharan Africa (SSA). Despite growing insights into HIV-associated immune ageing, there is still no universally applicable biomarker of immunosenescence; hence a need to develop robust, population-tailored biomarker panels and targeted interventions for PLHIV, to inform strategic interventions that combine ART with immune restoration and anti-ageing approaches, ultimately improving immune health and quality of life (15).

Currently, viral load is the main test for monitoring HIV treatment cohorts in SSA with less emphasis on CD4 counts, CD4/CD8 percentage and other potential markers of immune aging, immune function, and HIV-specific immune responses (16). Similarly, the extent to which HIV-1 reservoir characteristics such as size, cellular distribution, transcriptional activity, and clonal expansion correlate with immune aging phenotypes remains insufficiently defined in SSA where the HIV epidemic is characterized by heterogeneous non-B viral subtypes. This systematic review describes candidate biomarkers of immune aging during HIV treatment globally and their potential for clinical application in monitoring recovery of host HIV-specific immune responses, immune aging and risk of age-related NCD complications among adults aging with HIV and ART in SSA. The data provided herein informs the need to further understand, measure and monitor the phenomenon of immune aging among PLHIV in SSA, to inform strategic interventions to delay or reverse HIV-associated immune aging and the associated immunological and clinical consequences.

## Methods

On April 1, 2026, we searched PubMed using a broad Boolean strategy that combined MeSH terms and title/abstract keywords for HIV-related concepts, with MeSH terms and title/abstract keywords for immunosenescence-related concepts. The HIV block included terms such as HIV, HIV infections, HIV seropositivity, HIV-1, HIV-2, AIDS, and descriptors for people living with HIV, while the immunosenescence block included immunosenescence, T-cell senescence, cellular senescence, immune aging/ageing, inflammaging, age-associated immune changes, and accelerated or premature immune aging. The two concept blocks were combined with AND to identify studies addressing both HIV and immune aging. The PubMed Query used is in Supplement 1. For this systematic review, we limited the search to PubMed as this database is accessed for free, focuses mainly on the life sciences and biomedical disciplines and captures a large core of peer-reviewed biomedical journal literature that would be captured by other databases like Scopus, Web of Science, and Google Scholar, except for broader citation tracking (17). Next, the PubMed identifiers (PMIDs) obtained from the PubMed search were exported to a text file and used to retrieve the corresponding PubMed records in XML format using the efetch command from the NCBI Entrez Programming Utilities. We then used a custom Python script to parse the XML record and broadly categorize each article as clinical interventional, clinical observational, preclinical, or another distinct category outside these groups. We also extracted additional descriptive information, including publication year, title, and keywords, to support further refinement of the categorization during manual review. Lastly, we used another custom Python script to parse the affiliations of each author for each article, extracting author country and assigning continent. Together, these data provide a useful framework for assessing the types of studies identified, where the work was conducted, and which articles may warrant more detailed review and data extraction. During manual review, we filtered the resulting articles and only included articles that had primary data on biomarkers of immune aging including immune phenotypes, molecular markers, cytokines and proteomics data and reservoir quantification studies, among others. We also searched these for articles that analysed or reported any drivers of immune aging recorded. The findings were summarized and presented in **Tables 1**–**3**.

**Table 1:** Biomarkers of immune aging described during anti-retroviral therapy.

Citation	Cohort description	Immune aging biomarkers
Zanet et al. (19)	Canada, 395 participants, 76% female; 229 HIV-infected (median age: 40 years) and 166 HIV-uninfected (median age 38 years)	Median leukocyte telomere length (LTL) was significantly shorter in HIV + (2.9 kb/genome) than HIV-negative controls (3.0 kb/genome, p=0.03).
Ayala-Suárez et al. (38)	Spain; 52 male participants from cohorts with chronic HIV infection	SA-βGal (senescence-associated β-galactosidase), p16INK4a (cell cycle inhibitor) and γH2AX (DNA damage marker) were increased in CD4+/CD8+T-cells; Bcl-2(anti-apoptotic protein increased in CD4+ T cells
Kelesidis et al., (102)	USA, 234 ART-naïve adults treated for over 96 weeks.	T-cell Senescence markers; CD28−CD57+ CD4+ declined significantly by week 96 of ART
Tassiopoulos et al., (103)	USA, 1291 participants with median age 37 years, 96weeks of ART and 48 HIV negative controls	CD28 loss was used as a marker of senescence: %CD28−CD4+ T cells and %CD28−CD8+ T cells were elevated in untreated HIV. ART reduced CD8 senescence but did not normalize it
Affandi et al. (21)	Australia, 45patients (20HIV+CMV+, >50 years, ART for 12–17 years	The strongest marker of NK-cell immune aging was CD57 expression, which was highest in HIV patients
Shellard et al. (20)	Zambia and Zimbabwe. 842 participants adolescents aged 11–19 years from Zambia and Zimbabwe enrolled in the VITALITY trial	Shorter leukocyte telomere length (LTL) was observed in those with viral load (VL) >1000 copies/mL and lower CD4 counts; Persistent unsuppressed viremia led to significantly faster telomere attrition, equivalent to approximately 11 years of additional biological aging
Zhao et al., (104)	USA, 368 adult participants (183 HIV+, 185 age-matched healthy subjects), all HIV+ were adults on suppressive cART	CD57 expression was significantly higher in CD4 T cells; Telomere and chromosomal biomarkers (Telomere erosion & Telomeric DNA damage) were more pronounced in HIV+ CD4 T cells memory subsets, and inversely correlated with apoptosis; while DDR (DSB Sensors & Checkpoint Kinases) signaling was impaired
Leung et al. (2)	Canada, cohort of 31 participants who inject drugs mean age 35.8 ± 10.1 years, 48% male, 90% had hepatitis C coinfection	Absolute leukocyte telomere length (aTL) declined significantly after HIV acquisition (227 vs 201 kbp/genome, p=0.045), indicating rapid replicative senescence at seroconversion
Babu et al. (1)	India, 137 Participants: Median age: 45 years (IQR 42–49): Median ART duration: 8 years (IQR 6–10), Median CD4 count: 667 cells/µL	Telomere length was significantly shorter in PLHIV on ART compared with HIV-negative controls, independent of age and gender. Reservoir burden remained detectable, with median total HIV-1 DNA at 2.87 log10 copies/10^6^ PBMC, despite long-term suppression
Singh et al. (18)	USA, compared two clinical groups: 104 HIV+, Age: ≥45 years, cART for ≥1 year and 92 HIV-negative matched for cardiovascular risk using the Reynolds cardiovascular risk score	Intracellular Signaling and Molecular Regulators (p90RSK, NRF2, ERK1/2) were measured; Telomere length was shortened by ART, mediated by p90RSK activation, and restored by p90RSK inhibition (FMK-MEA) or NRF2 activation; Canonical senescence markers (p16 and p21) were elevated with ART but blocked by FMK-MEA and NRF2A, confirming p90RSK’s role in driving cellular senescence
Chen et al., (22)	China, 20 participants received 24 weeks of thymosin α1 (Tα1) plus ART. Male (19/20;95%), median age 48.1 years, median ART duration of 5.0 years (IQR 3.0–7.3), median baseline CD4 count of 215.3 cells/µL	Thymic output, measured by sjTREC, rose significantly over time despite a temporary decline at week 12, suggesting improved regenerative capacity. Naïve CD4 T cells expanded markedly, while central memory CD4 cells decreased, reducing reservoir-favoring subsets. Effector memory CD4 cells remained stable, but TEMRA CD4 cells increased, reflecting some terminal differentiation

PLHIV, people living with HIV; ART, antiretroviral therapy; γ-H2AX, phosphorylated histone H2AX; DDR, DNA Damage Response; DSB, DNA Double-Strand Break Sensors; ATM, Ataxia-telangiectasia mutated; CHK2, Checkpoint kinase 2; p90RSK, p90 Ribosomal S6 Kinase; NRF2, Nuclear Factor Erythroid 2-Related Factor 2; ERK1/2, Extracellular Signal-Regulated Kinase 1/2; p16INK4a, p16, cell cycle inhibitor; p21WAF1/CIP1, p21, cell-cycle arrest.

**Table 2:** Predictors of immune aging during ART.

Citation	Cohort description	Predictors of immune aging during suppressive ART
Heath et al. (25)	Canada, 134 (19HC) participants with a median age of 48(44) years, 15(13) median years of ART. 134/153 (88%) were CMV+, 19/153 (12%) CMV−.	Telomere length analysis showed progressive shortening across immune subsets: total lymphocytes > CD8+ T cells > CD57+CD8+ T cells > CMV-specific CD57+CD8+ T cells, confirming stepwise proliferative aging
Mueller et al. (27)	Germany, 92 Participants were matched 1:1:1:1 by age and sex into four groups (n=23/group): HIV+CMV+, HIV+CMV−, HIV−CMV+, and HIV−CMV−. Women: 30.4% (7/23 in each group), Median HIV suppression: HIV+CMV+: 64 months (IQR 20–96) HIV+CMV−: 62 months (IQR 15–108)	CMV exerted the strongest influence on CD8 senescence, driving expansion of late effector (CD27−CD28−) and terminally differentiated HIV altered terminal maturation by expanding transitional CD28−CD57− cells, suggesting incomplete terminal differentiation.
Kapetanovic et al. (26)	USA, 107 Children were grouped by baseline CMV status into: CMV+ viremic (detectable CMV DNA (≥12 copies/mL) n=15), CMV+ aviremic (no detectable CMV DNA n=52), and CMV-naïve (no evidence of CMV infection n=40). Median age: 7 years (IQR 4–10), Male: 55%	Terminal differentiation markers revealed CMV’s stronger impact on CD8 cells. CMV+ children had persistently higher frequencies of terminally differentiated CD8+CD95+CD28− and CD8+CD62L−CD45RA+ cells, with slower declines after ART compared to CMV-naïve.
Serrano-Villar et al. (33)	USA, 353 Participants from four established cohorts and two randomized clinical trials involving diverse HIV populations.	Senescence-associated phenotypes, including CD28− and CD57+CD8 T cells, were enriched in the low CD4/CD8 ratio group, alongside higher PD-1+ CD8 T-cell counts indicating exhaustion.
Martínez-Sanz et al. (34)	Spain, 4625 ART-treated participants median age of 37yr, (IQR 31–44), 3852 (83%) male ART duration 2 years	A CD4/CD8 ratio <0.3 at two years of ART was associated with increased risk of cardiovascular disease, non-AIDS malignancies, and non-accidental deaths over the following five years and CD4/CD8 ratio <0.4 were linked to increased SNAE risk.
Li et al., (105)	Retrospective MACS-based study 27 male PLHIV on suppressive ART and 10 HIV negative individual	Longitudinal analysis showed no significant changes in senescence-associated secretory phenotype (SASP) markers profiles over four years, suggesting stable but persistent chronic senescence signaling due to intact provirus and not defective reservoir as measured by matrix metalloproteinase-9 (MMP-9) level
Shive et al. (24)	USA,100 participants in three groups: Healthy controls (HC)/HIV-negative, n=20; Immune success (IS): ART-suppressed HIV+, CD4 >500/µL, n=20; & Immune failure (IF): ART-suppressed HIV+, CD4 <350/µL, n=60.	CD57+ senescent cells were increased in immune failures, especially in naïve and central memory compartments, highlighting accelerated senescence in populations that should remain proliferative.

**Table 3:** Association of immune aging with persistent viral reservoir during HIV treatment.

Citation	Cohort description	Association of immune aging with latent reservoir characteristics
Acharya et al. (29)	USA, 29 human participants with HIV and 14 SIV-infected, ART-treated rhesus macaques	Older individuals and aged macaques had significantly larger intact and total reservoirs, as well as higher levels of 3′ defective proviral DNA compared with younger counterparts, while 5′ defective proviral DNA showed no age-related differences
Royston et al. (28)	Canada, CIAO trial protocol (CMV Inhibition with Letermovir in ART-treated PLHIV, an Open-label, Randomized, Controlled Trial); ART duration ≥3 years	HIV reservoir biomarkers (HIV DNA, RNA, and inducible reservoir assays) confirmed residual viral persistence despite ART, with reservoir size linked to inflammatory and senescence profiles
Nottet et al. (14)	Netherlands, 8 participants, 8.3 years of ART	HIV-1 DNA load was significantly higher in aged memory CD4+ cells (CD45RO+CD57+) compared with non-senescent memory CD4+ cells
Domínguez-Rodríguez et al. (30)	England, Spain and Italy CARMA/EPICAL trial. 40 early-treated, perinatally HIV-infected children on long-term suppressive ART	Larger reservoirs displayed a vastly expanded percentage of terminally senescent CD4+CD28-CD57+T-cells, telomere shortening (with an Incidence Rate Ratio [IRR] of 0.15)
Casado-Fernández et al. (70)	Spain, 45 male participants, study compared ART-treated 21 PLHIV mpox-negative versus 24 ART-treated PLHIV mpox-positive; Median age 39.5 years; median HIV duration 9 years	Smaller reservoir was more inducible reservoir, especially in central memory (TCM) CD4+T-cells; with increased level of CD57+ T cells, reinforcing exacerbation of immune aging through viral co-infection
Martínez-Bonet et al. (31)	Spain, 242 participants, Duration on ART 10-17yrs Median age 18-20years	Strong inverse correlation between total HIV-1 DNA and percentage of memory CD4+ T cells in wild-type (CCR5 WT/WT) individuals, and a strong direct positive correlation in heterozygous (CCR5 WT/Delta32) individuals (maintained and sustained by homeostatic proliferation of memory T cell clones)
Dalzini et al. (11)	Italy; 37 perinatally HIV-infected children/adolescents (median age 13.8 years) who started ART start before age 2 years ART duration: at least 5 years.	Larger reservoir size consistently associated with accelerated telomere shortening, increased CD4 senescence (%CD28−CD57+) and reduced thymic regenerative capacity in CD8+ cells. Later ART (after 6 months but before 2 years) increased reservoir size by 6 fold, with reduced thymic output and 3 fold more CD4 senescence
Yang et al. (36)	Canada, 187 PLHIV (105 female/82 male) and 190 HIV-negative participants (105 female/84 male), ranging in age from 0.7 to 76.1 years	Women exhibited greater reservoir persistence, with more viral DNA than male counterparts; viral DNA was associated with increased senescence
Khanal et al. (5)	The study experiments were performed in two biological systems, i) -SupT1 cells (highly HIV-permissive T-cell line) & Primary human CD4 T cells& (isolated from healthy donors)	DDR markers (53BP1, γH2AX) and telomeric damage foci (TIFs via 53BP1/TRF1 colocalization) were significantly increased, indicating telomere-specific DNA damage. Telomere length was shortened in HIV-infected cells but partially restored with RAL.

PLHIV, People living with HIV; ART, antiretroviral therapy; γ-H2AX, phosphorylated histone H2AX; DDR, DNA Damage Response; RAL, Raltegrovir.

## Results

### Eligible articles

**Figure 1:**
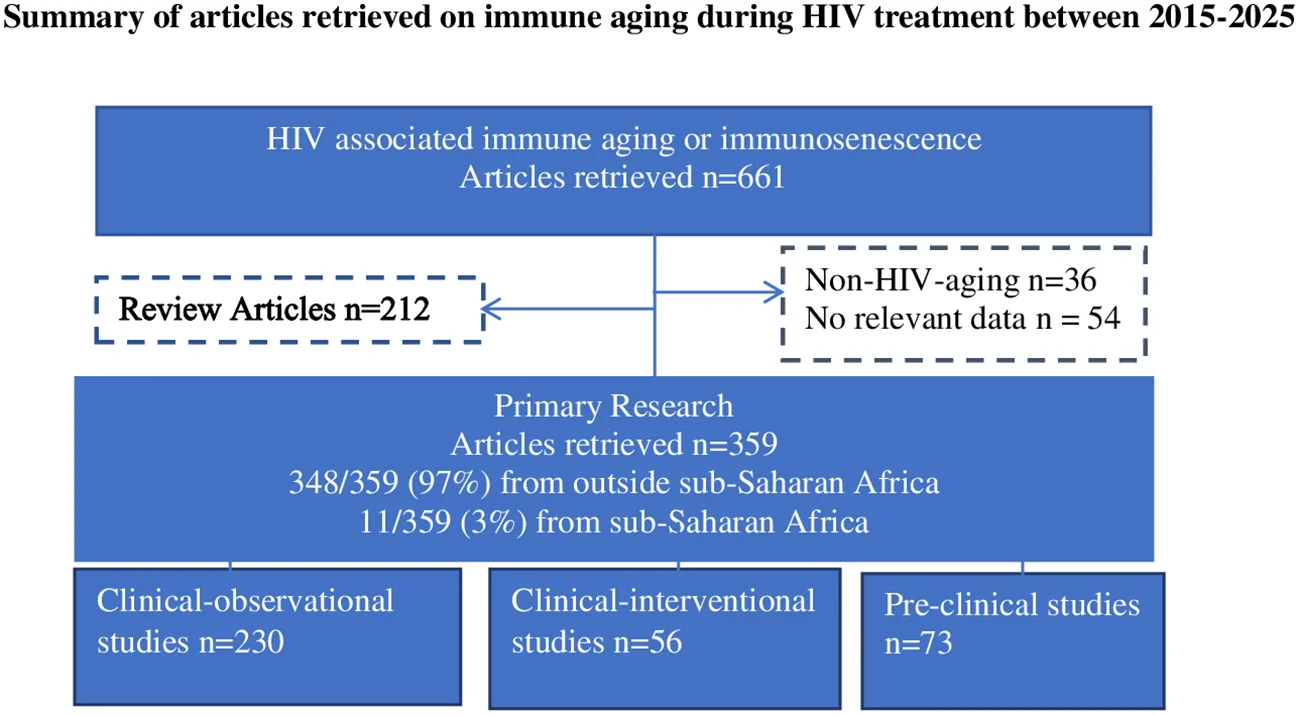
Summary of articles retrieved on immune aging during HIV treatment between 2015-2025.

As illustrated in the flow chart in **Figure 1**, we retrieved 661 articles. Of these, 36 articles that did not include HIV-infected individuals, 54 that did not have immune aging related data, and 212 review articles were excluded. Of the 359 studies that were included, only 11 (3%) were from SSA; and majority of the studies were observational studies 230/359 (64%), with only 56/359 (16%) as clinical intervention studies. The content reported below includes biomarkers of immune aging, drivers and correlation with HIV reservoir during HIV treatment.

### Biomarkers of immune aging described during HIV treatment

#### Telomere shortening

Across diverse cohorts globally, telomere shortening consistently emerged as a marker of HIV-accelerated immune aging, as outlined in **Table 1**. In India, telomere shortening persisted despite ART and was linked to reservoir burden, with inflammatory mediators such as CXCL1 and TGF-α associated with longer telomeres, while IL-10RA correlated negatively (1). Complementary mechanistic studies in U.S. cohorts showed oxidative stress–driven p90RSK activation shortened telomeres and promoted vascular aging, reversible by p90RSK inhibition or NRF2 activation (18). Similarly, HIV^+^ CD4 cells displayed telomere attrition, elevated ROS, DNA damage markers (γH2AX, p53, TIFs), and impaired ATM/CHK2 repair signaling, linking oxidative stress and defective DNA repair to persistent immune dysfunction despite viral suppression (6). A couple of studies in African adults showed that shorter leucocyte telomere length (LTL) correlated with higher peak HIV RNA levels and Hepatitis C virus (HCV) co-infection but not with ART exposure or viral suppression (2, 19) and persistent viremia increased biological aging by approximately 11 years relative to viral suppressed peers (20).

#### Immune phenotyping

Immune phenotyping revealed that CD28⁻CD57^+^ CD8^+^ T-cells, a hallmark of CD8 senescence, remained largely unchanged across regimens. In contrast, CD28⁻CD57^+^ CD4^+^ T-cells and PD-1^+^ subsets declined significantly, demonstrating capacity of ART to reverse CD4 senescence and exhaustion albeit partially. NK cells shifted toward terminally differentiated, cytotoxic CD56^lo perforin^hi phenotypes with loss of cytokine-producing CD56^hi subsets (21). In a Chinese cohort, 24 weeks of Tα1 alongside ART enhanced thymic output (sjTREC), expanded naïve CD4^+^ T cells, reduced reservoir-prone central memory subsets, and lowered PD-1 expression on CD8^+^ cells (22, 23). Mechanistic studies confirmed that IL-1β and IL-7 stimulation directly induced PD-1 and CD57 expression on healthy CD4 T cells, connecting inflammatory signaling and disrupted homeostasis to exhaustion and senescence. In contrast, healthy controls maintained intact IL-7/CD127 regulation, low PD-1/CD57 expression, and high Bcl-2, reflecting preserved immune homeostasis (24). Despite modest absolute CD4 recovery, Tα1 shifted T-cell composition toward a less senescent, less reservoir-favoring phenotype in immunological non-responders (6).

### Drivers of immune aging during ART

Cytomegalovirus (CMV) co-infection, persistent microbial translocation, oxidative stress, and chronic immune activation are drivers (mechanistic forces which push the immune system towards senescence) of immune aging in PLHIV, included in **Table 2**. CMV co-infection exacerbates immune aging among PLHIV through systemic inflammation and expansion of late effector (CD27⁻CD28⁻) and terminally differentiated CD57^+^CD95^+^CD62L⁻CD45RA^+^ CD8 T-cells. CMV-specific CD57^+^CD8^+^ T-cells consistently showed the shortest telomeres even shorter than HIV specific CD8^+^ T cells underscoring CMV as a dominant driver of replicative exhaustion. CMV-infected individuals also exhibited lower CD4/CD8 ratios, linking senescence to systemic inflammation and highlighting CMV’s central role in long-term immune aging (25–27). Innate immune dysfunction was evident in NK cells, where CD57 expression was highest in HIV patients compared with CMV^+^ and CMVs⁻ controls, indicating HIV as the dominant driver of NK-cell senescence. A recent study integrating microbial translocation, systemic inflammation, monocyte/T-cell activation, CMV responses, HIV reservoirs, and microbiota composition found that ART suppressed HIV infection remains characterized by persistent microbial translocation, chronic inflammation, immune activation and CMV driven senescence converging into an inflammaging profile (28).

### Association of immune aging with persistent HIV viral reservoir

Emerging evidence summarized in **Table 3** demonstrates that persistent HIV reservoir is likely associated with accelerated immune aging among PLHIV. In a U.S. study of 29 ART-treated participants, immunosenescence was associated with clonal expansion of infected memory CD4^+^ T cells (29). Complementary findings from the Netherlands revealed that senescent terminally differentiated memory CD4^+^ T-cells serve as preferential reservoirs for HIV persistence (14). Evidence from perinatally infected children and adolescents in Europe (Spain, Italy, and England) further showed that ART initiation within the first six months of life resulted in smaller reservoir sizes, preserved telomeres, and minimal accumulation of senescent cells. In contrast, delayed initiation (after six months but before two years) led to significantly larger reservoirs and increased CD4^+^ T-cell senescence (11, 30). Collectively, these findings demonstrate that even brief periods of unchecked viral replication in early infancy can seed a durable persistent reservoir that may be associated with premature immune aging.

We also reported association of host genetic traits with immune aging and persistent HIV viral reservoir during HIV treatment. In individuals with the CCR5WT/WT genotype, a shift toward memory T-cell phenotypes was associated with smaller reservoir sizes, whilst in CCR5WT/Δ32 carriers more differentiated memory T-cell profiles translated into larger detectable reservoirs in peripheral blood (31). Cells harboring HIV-1 DNA displayed shorter telomeres and elevated telomeric DNA damage compared to healthy controls. Integrated viral DNA correlated with sustained activation of the ATM–p53–p21 signaling pathway, pushing otherwise healthy CD4^+^ T-cells into premature senescence and halting their proliferation. In larger HIV reservoir states, protective PI3K signaling was blunted while the ATM pathway remained hypersensitized, creating a molecular imbalance where aged CD4^+^ T-cells with persistent oxidative or metabolic stress bypassed repair mechanisms and underwent apoptosis (5). Noteworthy, is the limited availability of data on HIV-associated immune aging and the persistent HIV reservoir within HIV treatment cohorts SSA.

## Discussion

This systematic review exposes the scarcity of well-characterized evidence of critical HIV-associated immune aging biomarkers in SSA despite three decades of the epidemic in the region. We therefore focus our discussion on evidence generated in the global north regarding potential markers of immune aging and related pathways, that can be investigated for adoption in HIV treatment cohorts in SSA. In general, cellular senescence and inflammaging emerge as drivers of age-related dysfunction; more so among PLHIV where these may affect the host immune responses required for durable HIV-1 viral clearance. Common markers of dysfunction including senescence-associated β-galactosidase (SAβGal) activity at pH 6.0, elevated levels of p16INK4a/p21CIP1, SASP, and telomeric dysfunction are present in aged human CD8+ T cells (32). Moreover, senescent cells can exhibit diverse features such as morphological and molecular changes that result in stable cell cycle arrest and resistance to apoptosis, and the constituent senescence-associated secretory phenotype (SASP) (7) that need evaluation during long-term HIV treatment.

### Biomarkers of immune aging during long-term HIV treatment

There was no single universal biomarker of HIV-associated immunosenescence globally. Overall, CD4/CD8 ratio emerged as a robust biomarker of immune aging in several cohorts. In a U.S. cohort of 353 participants across multiple studies, a persistently low ratio identified individuals with chronic immune dysfunction, inflammation, and immunosenescence, and a higher risk of non-AIDS events and mortality (33). Persistent inversion of the CD4/CD8 ratio and elevated CD8 counts may therefore serve as key markers of immune aging and predictors of serious non-AIDS outcomes (34). Similarly, a CD4/CD8 ratio <0.3 at two years of ART predicted increased risk of age-related comorbidity including cardiovascular disease, non-AIDS malignancies, and non-accidental deaths over the next five years (OR 1.63, 95% CI 1.03–2.58), with an even stronger effect at <0.2 (OR 3.09, 95% CI 1.57–6.07). CD4/CD8 ratio is one of the most clinically validated measures of immune aging because it is inexpensive, routinely measured, reproducible across cohorts, and consistently associated with non-AIDS morbidity and mortality. However, the ratio lacks specificity because it may also be influenced by CMV co-infection, chronological age, duration of ART, and other factors unrelated to immune senescence. Therefore, while the CD4/CD8 ratio remains an attractive biomarker for large-scale clinical monitoring, it may be most informative when interpreted alongside additional markers of immune activation, inflammation, and cellular senescence. Furthermore, high CD8 counts alone were even stronger than CD4/CD8 ratio in forecasting AIDS and non-AIDS outcomes, while low CD8 counts predicted severe infections (35). Moreover, women living with HIV experienced a more amplified rate of biological aging compared to uninfected women, while this effect was less pronounced in men (36). Although PD-1 is widely used as a marker of T-cell exhaustion, its utility as a standalone biomarker of immune aging remains uncertain. PD-1 expression may reflect ongoing antigen exposure and immune activation rather than irreversible senescence. Furthermore, substantial variability exists across studies regarding the cell populations evaluated and the thresholds used to define PD-1 positivity. Consequently, PD-1 may be most informative when interpreted alongside established markers of senescence such as CD57 expression, loss of CD28, telomere shortening, and alterations in the CD4/CD8 ratio. Currently, there is not enough evidence on the biomarkers in African cohorts to advise the exact immune aging biomarkers (singly or in combination) that could constitute either predictive, diagnostic or monitoring panels for HIV-associated immune aging and its complications among PLHIV in SSA. Hence, a need to evaluate immunological characteristics of exhausted and senescent cells that include markers of reversible cell cycle arrest, increased expression of LAG3, PD-1 and TIM3, as well as reduced IL-2 and Inteferon production. Similarly, markers of irreversible cell cycle arrest such as increased expression of CD57 and KLRG-1, reduced CD28, increased SASP, increased SA-βGal activity, and increased telomeric erosion (7) that contribute to defective effector functions need to be evaluated in HIV treatment cohorts in SSA.

ART-treated PLHIV demonstrate terminal differentiation, immune exhaustion, chronic inflammatory signaling, altered monocyte and macrophage activation, telomere attrition, and epigenetic or transcriptional signatures that are consistent with accelerated aging (1, 37–39). These alterations directly influence ability of the host immune system to recognize infected cells, expand in response to antigenic stimulation, and sustain effective effector or memory responses. Multiple studies identify signatures of T-cell senescence, including CD57 expression, CD28 loss, terminal differentiation, inflammescent phenotypes, and transcriptional profiles associated with reduced functional reserve (4, 36, 40, 41). Thus, there is a need for well-characterized longitudinal evidence on the association of these immune aging biomarkers with long-term outcomes of HIV treatment in SSA, where current monitoring of long-term HIV treatment is focused on viral load measurement, without routine CD4 counts or any other measures of immune senescence and immune function recovery.

There is increasing evidence to suggest that accumulation of inflammatory burden over time increases oxidative stress and disrupts tissue repair mechanisms, thereby accelerating the aging process and increasing the risk of various chronic diseases (42). For example, dysfunction of cytoplasmic Pattern Recognition Receptors (PRRs) (e.g., retinoic acid-inducible gene I) impairs downstream type I interferon (IFN-I) signalling, resulting in diminished phagocytic capacity and blunted IFN-I responses (43), that indicate a broader dysfunction of the mitochondrial–PRR axis and are closely linked to immunosenescence. Similarly, epigenetic remodelling, such as elevated SET domain-containing 7 expression and increased H3 lysine 4 monomethylation at proinflammatory gene promoters (e.g., NF-κB p65), sustains monocyte proinflammatory programming (44). Similarly, in aging individuals, increased CD86 expression in macrophages reflects a shift towards the M1 phenotype, characterized by heightened proinflammatory activity (45). In animal models of liver injury, aging amplifies M1-polarized responses and increases the expression of SASP factors such as IL-6 and TNF-α (46). Paradoxically, aging can also promote M2 polarization in certain contexts, leading to impaired resolution of chronic inflammation and diminished immune surveillance. Key signaling pathways that regulate the SASP, such as the NF-κB, mTOR, p38 MAPK, and JAK/STAT pathways, have been recognized as potential drug intervention targets (42), and these could potentially be expanded to clinical trials in non-B HIV sub-type infections in SSA. Generation of local evidence on the spectrum of markers of immune aging, immune exhaustion, and general inflammation; and how these may play out in the SSA settings of heterogeneity in host, HIV viral sub-types, endemic co-infections, and indigenous diets, among other factors, is required to guide the development of predictive and/or diagnostic and/or monitoring biomarker panels for HIV-associated immune aging in SSA. Once developed, such panels would then be tested for scale-up and cost-effectiveness within the clinical care and laboratory settings in SSA; and more so for the development of point-of-care diagnostics that would optimize the cost, scalability and sustainability.

### Association of immune aging biomarkers of NCD during HIV treatment

Data from high income countries illustrates that immune aging phenotypes were linked to carotid plaque, arterial inflammation, blood pressure, metabolic dysregulation, cognitive decline, cancer risk, and broader non-AIDS outcomes (1, 4, 9, 10, 47–49), although there is limited evidence to support these associations and mechanistic data representative of cohorts in SSA. Immunosenescence, marked by CD28^−^CD57^+^ T-cell accumulation, affects treatment outcomes in PLHIV. The degree of immune aging partly predicts the effectiveness of immune recovery, with low CD4 counts linked to increased CD28^−^CD4^+^ and CD28 CD8 + ratios (50). The accumulation of CD27⁻CD28⁻ CD8^+^ T cells is a recognized hallmark of immunosenescence. CD27⁻CD28^+^ terminally differentiated effector memory RA+ (TEMRA) cells have been identified as strong predictors of all-cause mortality in older individuals, with the CD27⁻CD28⁻ subset exhibiting the shortest telomere lengths (51, 52). Moreover, CD8^+^CD28⁻ T cells exhibit a canonical immunosenescent phenotype and have prognostic value in AIDS-related non-Hodgkin lymphoma (40). Such data is required to understand the predictors, mechanisms and complications of HIV-associated immune aging during HIV treatment, particularly in SSA where the HIV epidemic in SSA is characterized by heterogeneity of HIV sub-types and endemic co-infections that likely influence host immune function recovery during HIV treatment.

### Drivers of immune aging during suppressive HIV treatment

Noteworthy in this review is the fact that HIV-associated immune aging and its drivers are largely modifiable through interventions that target inflammation, immune restoration, and co-infections. These include thymosin α1 in immunological non-responders, omega-3/fish oil interventions, statin-associated reversal of cellular senescence effects, letermovir-based reduction of gut inflammation, and modulation of cytokine pathways such as IL-7, IL-15, and IL-21 (22, 28, 53–57). Although these studies do not establish a standardized clinical pathway, they indicate that HIV-associated immunesenescence can be delayed or reversed through adjunctive interventions that reduce inflammation, manage co-infections, restore immune function, and limit NCD risk before deploying cure directed strategies. The strongest direct Africa-focused evidence includes studies from Uganda, South Africa, Tanzania, Mozambique, and broader African cohorts that highlight immune aging burden during HIV treatment (3, 58–62). These studies are still too few relative to the scale of the epidemic, but they indicate concern that immune aging has clinical consequences in SSA. Similarly, co-infections such as CMV, helminths, Leishmania, malaria, tuberculosis, among others, that potentially amplify immune activation and senescence, underscore the need to understand immune aging and HIV cure in SSA within a broader ecological and infectious context (59, 63–65). Furthermore, with the HIV epidemic in SSA predominantly affecting females, further research is needed to understand the role of biological sex in HIV-associated immune aging among males and females (66, 67).

### Immune aging and the persistent HIV reservoir during long-term HIV treatment

Despite durable viral suppression with ART, the persistent HIV reservoir that is replication-competent remains a critical barrier to HIV cure as it can lead to viral rebound upon ART interruption (68). Understanding the fine-tuned interplay between host immune responses and viral reservoir cells will help to design improved interventions that exploit the immunological vulnerabilities of HIV-1 reservoir cells (69). Immune aging and latent HIV reservoir size appear to reinforce one another. Several studies demonstrate association of transcriptionally active persistent HIV reservoir with senescence pathways including cytokine patterns, telomere shortening, and age-related reservoir expansion (11, 12, 14, 29, 30, 70). Even if transcriptionally restricted, the persistent HIV reservoir may still contribute to low-level antigenic stimulation, inflammatory signaling, and immune activation that promote progressive immune aging. Conversely, immune aging may help maintain the reservoir by reducing immune surveillance and facilitating clonal persistence of infected cells. This bidirectional relationship needs to be investigated particularly in SSA, where delayed ART initiation likely permits both deeper reservoir establishment and greater cumulative immune injury before virological control is achieved. Furthermore, persistent reservoirs may drive telomere attrition, senescence, and exhaustion, while factors such as CCR5 genotype, sex differences, and viral co-pathogens further modulate this relationship (36).

### Implications of immune aging for potential HIV cure interventions in SSA

The studies reviewed herein demonstrate that a senescent or terminally differentiated immune compartment may still be numerically present, but less adaptable, less proliferative, and less capable of sustained cytotoxic function. Hence a potential risk for “shock and kill,” HIV cure strategies which depend not only on induction of viral expression but also on subsequent immune recognition and clearance. Furthermore, telomere attrition, DNA damage signaling, mitochondrial dysfunction, and epigenetic aging were reported in both adult and pediatric studies, including a couple of African cohorts (2, 5, 6, 20, 59, 71, 72). These findings imply reduced regenerative capacity of the immune system that, if unaddressed, makes hosts less likely to sustain effective vaccine responses, cytotoxic bursts, or durable post-treatment immune control. Consequently, ART treated individuals with immune aging are likely to exhibit blunt responsiveness to therapeutic vaccines (60, 73, 74) suggesting that vaccine-based cure approaches may underperform in these sub-populations of PLHIV. However, the block-and-lock strategies may be relatively more attractive in immune aged populations because they depend less on vigorous immune clearance and more on durable suppression of viral transcription. Although there is limited direct clinical evidence for block-and-lock outcomes in SSA, the biological rationale is strengthened by the persistence of Tat-associated DNA methylation changes and latency modulation (75–77). Therefore strategies to rejuvenate an aged immune system and restore immune resilience should be considered among PLHIV in SSA; including cell-based therapies (Chimeric Antigen Receptor T Cell (CAR-T cells); CTLA-4: Cytotoxic T Lymphocyte Associated Protein-4; iPSC: Induced Pluripotent Stem Cells; PD-1: Programmed Cell Death Protein 1), and pharmacological approaches that have been demonstrated to restore immune function in mice and humans (7, 78).

A functional cure could be achieved by durably silencing the latent provirus in infected cells and thereby preventing viral rebound (79), through the so-called “block-and-lock” approach to prevent HIV transcription and reactivation in latently infected cells. “Block and lock” strategies aim to suppress residual levels of HIV-1 transcription to lock the virus in deep latency state to prevent viral reactivation (80–82) and permanently silence all proviruses, even after treatment interruption. HIV silencing can be achieved by targeting different Trascription Factors (TFs) of the transcription machinery. TFs are cellular and viral proteins that bind to specific DNA/RNA sequences in the HIV long terminal repeat (LTR) promoter to control gene expression and latency. They dictate whether the provirus remains dominant or actively replicates. Nuclear factor kappa B (NF-κB) is a transcription factor involved in T cell activation that activates HIV transcription even in the absence of Tat (83), and a key cellular TF that binds the LTR enhancer region upon immune cell activation, initiating basal-host-cell level transcription AP-1 and NFkB synergies to transcriptionally activate latent HIV upon T-cell receptor activation (84, 85). Similarly, Sp1 and AP-1 are cellular TFs that constitutively bind the core promoter, maintaining baseline transcription activity. However, in resting cells NF-κB is sequestered in the cytoplasm by inhibitors of NF-κB (IκB). Nuclear factor of activated T-cells (NFAT), another transcription factor, is phosphorylated and resides in the cytoplasm of resting cells (86, 87). NF-κB and NFAT are two key factors for initiation of HIV transcription (82). Similarly, DNA methylation at CpG dinucleotides in the LTR promoter contributes to latency and restricts reactivation in cell lines and patient samples by hampering the access of transcription factors to the DNA (88, 89). Next to a block in transcription initiation or elongation, additional blocks may exist at the level of distal transcription and multiple splicing as postulated by the Yukl group (90). Moreover, there is evidence to suggest that blocks at transcription elongation, completion and RNA splicing may be tissue specific (82). For example, suboptimal concentrations or post-translational modifications of Tat hamper Tat-induced transcription elongation and phosphorylation of Tat by CDK2 results in inhibition of transcription (91). Tat and bromodomain containing protein 4 (BRD4) can release P-TEFb by disrupting the inactive complex (92). BRD4 represses HIV transcription by competing with Tat for the P-TEFb binding site (92, 93). Transcription is also affected by the absence or presence of host transcription factors and transcription repressors.

With the identification of the first block and lock HIV-1 therapeutic in 2005, the field has rapidly expanded the number of potential anti-HIV-1 therapeutics. Currently the most advanced block-and-lock approach to silence HIV transcription uses a Tat inhibitor, didehydro-cortistatin A (dCA), which inhibits Tat-dependent transcription and induces a repressive epigenetic landscape that hampers HIV reactivation upon treatment interruption (82, 84).This small molecule inhibitor progressed to *in-vivo* humanized bone marrow, liver, thymus (BLT) mouse studies in 2017, which demonstrated that dCA treatment delayed virus rebound until day 19 compared to day 10 in control mice (94). Another regulator of HIV transcription is the ‘facilitates chromatin transcription complex’ (FACT) that consists of suppressor of Ty16 (SUPT16H) and structure-specific recognition protein (SSRP1) (95). FACT acts as a histone chaperone and promotes RNAPII driven transcription by destabilizing the nucleosomal structure (96). There is evidence to suggest that curaxins can promote HIV latency via inhibition of FACT; for example, Curaxin CBL0100 inhibited RNAPII mediated transcription elongation in a Tat-dependent manner (97). However, the effect was independent of NF-κB binding to the 50 LTR promoter. It is likely that addition of CBL0100 to ART regimen might lead to a faster control of viremia and reduced HIV reactivation that eventually locks the virus in a latent state even upon treatment interruption (97, 98). Furthermore, HIV transcription could be silenced by using short interfering (si) or short hairpin (sh) RNA to maintain the repressive heterochromatic landscape at the HIV 50 LTR promoter. The Kelleher group designed two siRNAs, 143 and Prom A, which target transcription factor binding sites in the LTR promoter (99, 100). These siRNAs epigenetically silence HIV transcription by recruiting Argonaute 1 (AGO1), histone deacetylase 1 and histone methyl transferases (101). AGO1 is an essential component of the RNA-induced silencing complex (RISC) that binds siRNAs and cleaves the mRNA, a process termed RNA interference (RNAi). Both siRNAs reduced reactivation of the latently infected J-Lat cells by two-three-fold when challenged by different LRAs (101). The siRNAs could be delivered to ART-treated patients via retroviral vector transduced autologous CD4+ T cells or CD34+ cells in the absence of ART (99). However, further extensive preclinical evaluation is required. Most of these data is from *in-vitro* studies and *in-vivo* studies humanized mouse model in global North (84), and has been largely based in HIV-1 subtype B. Nevertheless, these data underscore the need for well-characterized investigation of all steps involved in HIV viral gene expression and nuclear topography of HIV which affect viral transcription (82) and human clinical trials of candidate transcription inhibitor molecules such as dCA amongst the heterogenous non-B HIV viral subtypes in SSA. Further, whether a combination of potentially shock and kill latent reservoir cells that can be reactivated, with block and lock the remaining latent reservoir cells to ensure permanent HIV-1 latency (84) may work best for chronic non-B HIV viral subtypes in SSA is yet to be investigated.

## Conclusion

This systematic review underscores the need for evidence to develop HIV-associated immune aging biomarker panels during long-term HIV treatment in SSA; to guide the development of predictive, diagnostic and/or monitoring biomarker panels for HIV-associated immune aging and its complications among PLHIV in SSA. We recommend well-characterized human studies to inform the adoption of context-specific HIV cure innovations in consideration of the heterogeneous host immune aging phenotypes, HIV-1 viral sub-types and endemic co-infections in SSA.
