# Supplementary material for: HIV associated immune aging among people living with HIV: implications for long-term antiretroviral therapy and potential HIV cure interventions in sub-Saharan Africa
Source: Front Immunol. 2026 Sep 3;17:1918867. doi: 10.3389/fimmu.2026.1918867 (PMC13581882; doi:10.3389/fimmu.2026.1918867)
Supplement: Supplementary file 1 [file Table1.docx]

| **Citation** | **Cohort description** | **Immune aging Biomarkers** |
| --- | --- | --- |
| Zanet et al., 2014 | Canada, 395 participants, 76% female; 229 HIV-infected adults (median age: 40 years) and 166 HIV-uninfected adults (median age 38 years) | Median leukocyte telomere length (LTL) was significantly shorter in HIV + (2.9 kb/genome) than HIV-negative controls (3.0 kb/genome, p=0.03), and remained shorter in multivariable analysis. |
| Ayala-Suárez et al., 2026 | Spain; 52 male participants from cohorts with chronic HIV infection | SA-βGal (senescence-associated β-galactosidase), p16INK4a (cell cycle inhibitor) and γH2AX (DNA damage marker) were increased in CD4+/CD8+T-cells; Bcl-2(anti-apoptotic protein increased in CD4+ T cells |
| Kelesidis et al., 2016 | USA, 234 ART-naïve adults treated for over 96 weeks. | T-cell Senescence markers; CD28−CD57+ CD4+ declined significantly by week 96 of ART |
| Tassiopoulos et al., 2012 | USA, 1291 participants with median age 37 years, 96weeks of ART and 48 HIV negative controls | CD28 loss was used as a marker of senescence: %CD28−CD4+ T cells and %CD28−CD8+ T cells were elevated in untreated HIV. ART reduced CD8 senescence but did not normalize it |
| Affandi et al., 2015 | Australia, 45participants (20 HIV+, CMV+, >50 years, on ART for 12–17 years, | The strongest marker of NK-cell immune aging was CD57 expression, which was highest in HIV patients compared with both CMV+ and CMV− controls |
| Shellard et al., 2026 | Zambia and Zimbabwe. 842 participants adolescents aged 11–19 years from Zambia and Zimbabwe enrolled in the VITALITY trial | Shorter leukocyte telomere length (LTL) was observed in those with viral load (VL) >1000 copies/mL and lower CD4 counts; Persistent unsuppressed viremia led to significantly faster telomere attrition, equivalent to approximately 11 years of additional biological aging |
| Zhao et al., 2019 | USA, 368 adult participants (183 HIV+, 185 age-matched healthy subjects), all HIV+ were adults on suppressive cART | CD57 expression was significantly higher in CD4 T cells; Telomere and chromosomal biomarkers (Telomere erosion & Telomeric DNA damage) were more pronounced in HIV+ CD4 T cells memory subsets, and inversely correlated with apoptosis; while DDR (DSB Sensors & Checkpoint Kinases) signaling was impaired |
| Leung et al., 2017 | Canada, longitudinal study, included 31 participants who inject drugs mean age 35.8 ± 10.1 years, 48% male, 90% had hepatitis C coinfection | Absolute leukocyte telomere length (aTL) declined significantly after HIV acquisition (227 vs 201 kbp/genome, p=0.045), indicating rapid replicative senescence at seroconversion |
| Babu et al., 2019 | India, 137 Participants: Median age: 45 years (IQR 42–49): Median ART duration: 8 years (IQR 6–10), Median CD4 count: 667 cells/µL | Telomere length was significantly shorter in PLHIV on ART compared with HIV-negative controls, independent of age and gender. Reservoir burden remained detectable, with median total HIV-1 DNA at 2.87 log10 copies/10⁶ PBMC, despite long-term suppression |
| Singh et al., 2019 | USA, compared two clinical groups: 104 HIV+ , Age: ≥45 years, cART for ≥1 year and 92 HIV-negative matched for cardiovascular risk using the Reynolds cardiovascular risk score | Intracellular Signaling and Molecular Regulators ( p90RSK, NRF2, ERK1/2) were measured; Telomere length was shortened by ART, mediated by p90RSK activation, and restored by p90RSK inhibition (FMK-MEA) or NRF2 activation; Canonical senescence markers (p16 and p21) were elevated with ART but blocked by FMK-MEA and NRF2A, confirming p90RSK’s role in driving cellular senescence |
| Chen et al., 2024 | China, 20 participants received 24 weeks of thymosin α1 (Tα1) plus ART. Male (19/20;95%), median age 48.1 years, median ART duration of 5.0 years (IQR 3.0–7.3), median baseline CD4 count of 215.3 cells/µL | Thymic output, measured by sjTREC, rose significantly over time despite a temporary decline at week 12, suggesting improved regenerative capacity. Naïve CD4 T cells expanded markedly, while central memory CD4 cells decreased, reducing reservoir-favoring subsets. Effector memory CD4 cells remained stable, but TEMRA CD4 cells increased, reflecting some terminal differentiation |

**PLHIV** (people living with HIV), **ART** (antiretroviral therapy), **γ-H2AX** (phosphorylated histone H2AX);**DDR** (DNA Damage Response), **DSB** (DNA Double-Strand Break) Sensors; **ATM** (Ataxia-telangiectasia mutated); **CHK2 (**Checkpoint kinase 2); **p90RSK** (p90 Ribosomal S6 Kinase): **NRF2** (Nuclear Factor Erythroid 2-Related Factor 2): **ERK1/2** (Extracellular Signal-Regulated Kinase 1/2); **p16INK4a** (p16) (cell cycle inhibitor); **p21WAF1/CIP1** (p21) (cell-cycle arrest).

**Supplement 1**

Pubmed query used to search for articles on HIV-associated immune aging:

("HIV"[MeSH Terms] OR "HIV Infections"[MeSH Terms] OR "HIV Seropositivity"[MeSH Terms] OR "HIV"[Title/Abstract] OR "HIV-1"[Title/Abstract] OR "HIV-2"[Title/Abstract] OR "human immunodeficiency virus"[Title/Abstract] OR "AIDS"[Title/Abstract] OR "PLWH"[Title/Abstract] OR "PWH"[Title/Abstract] OR "people living with HIV"[Title/Abstract]) AND ("Immunosenescence"[MeSH Terms] OR "T-Cell Senescence"[MeSH Terms] OR "Cellular Senescence"[MeSH Terms] OR "immunosenescen*"[Title/Abstract] OR "immune senescen*"[Title/Abstract] OR "immune aging"[Title/Abstract] OR "immune ageing"[Title/Abstract] OR "immunologic aging"[Title/Abstract] OR "immunological aging"[Title/Abstract] OR "immunological ageing"[Title/Abstract] OR "inflammag*"[Title/Abstract] OR "age associated immune*"[Title/Abstract] OR "accelerated immune aging"[Title/Abstract] OR "accelerated immune ageing"[Title/Abstract] OR "premature immune aging"[Title/Abstract] OR "premature immune ageing"[Title/Abstract] OR "t cell senescen*"[Title/Abstract] OR "t cell senescen*"[Title/Abstract] OR "aging immune system"[Title/Abstract:~2] OR "ageing immune system"[Title/Abstract:~2]).
